# 

**Published:** 1868-01-01

**Authors:** 


*CONTENTS:*

Pathological Physiology. The Continuous Venous Murmurs in the Neck. Translated by Walter Hay, M.D. .... 1
International Medical Congress .... 14
After Treatment in Necrosis. By Prof. Gunn .... 17

BOOK NOTICES:
Mechanical Therapeutics. By Philip Wales, M.D. .... 20
A Biennial Retrospect of Medicine and Surgery. By Mr. H. Power and Dr. Austee .... 21
On Diseases of the Lungs and Air Passages. By Henry William Fuller, M.D. .... 21
The Philadelphia Reporter .... 21
Ophthalmiatrische Beobachtungen. By Albert Mooren, M.D. .... 22
Spermatorrhœa. By Roberts Bartholow, M.D. .... 23
Pennsylvania Hospital Reports .... 23
Diseases of the Heart. By David Wooster, M.D. .... 23
A Practical Treatise on the Diseases of Children .... 23
Obstetric Clinic. By G. T. Elliott, Jr., M.D. .... 23
Hysteria. By F. C. Skey, F.R.S. .... 23
Report of the Wisconsin State Insane Hospital .... 23

LOOT:
Rhus Toxicodendron .... 24
Gonorrhœal Rheumatism .... 24
Bright's Disease .... 24
Abortive Treatment of Typhoid Fever .... 25
Brown Cod Liver Oil .... 25
Mammary Cancer .... 25
Acetic Acid .... 26
Permanganate of Potash .... 26
Sulphuric Ether in Cases of Tape Worms .... 26
Nutrition .... 27
Pepsine in Vomiting of Pregnancy .... 27

EDITORIALS:
The Twenty-fifth Volume .... 28
To Correspondents .... 29
Proceedings of Medical Societies .... 29
The International Congress .... 29
The Present Raid on the Uterus .... 30
Rush Medical College .... 31
Jucunde .... 31
The Next Issue .... 32

